# The G protein-coupled receptor subset of the rat genome

**DOI:** 10.1186/1471-2164-8-338

**Published:** 2007-09-25

**Authors:** David E Gloriam, Robert Fredriksson, Helgi B Schiöth

**Affiliations:** 1 Department of Neuroscience, Uppsala University, BMC, Box 593, 751 24, Uppsala, Sweden

## Abstract

**Background:**

The superfamily of G protein-coupled receptors (GPCRs) is one of the largest within most mammals. GPCRs are important targets for pharmaceuticals and the rat is one of the most widely used model organisms in biological research. Accurate comparisons of protein families in rat, mice and human are thus important for interpretation of many physiological and pharmacological studies. However, current automated protein predictions and annotations are limited and error prone.

**Results:**

We searched the rat genome for GPCRs and obtained 1867 full-length genes and 739 pseudogenes. We identified 1277 new full-length rat GPCRs, whereof 1235 belong to the large group of olfactory receptors. Moreover, we updated the datasets of GPCRs from the human and mouse genomes with 1 and 43 new genes, respectively. The total numbers of full-length genes (and pseudogenes) identified were 799 (583) for human and 1783 (702) for mouse. The rat, human and mouse GPCRs were classified into 7 families named the *Glutamate, Rhodopsin, Adhesion, Frizzled, Secretin, Taste2 and Vomeronasal1 *families. We performed comprehensive phylogenetic analyses of these families and provide detailed information about orthologues and species-specific receptors. We found that 65 human *Rhodopsin *family GPCRs are orphans and 56 of these have an orthologue in rat.

**Conclusion:**

Interestingly, we found that the proportion of one-to-one GPCR orthologues was only 58% between rats and humans and only 70% between the rat and mouse, which is much lower than stated for the entire set of all genes. This is in mainly related to the sensory GPCRs. The average protein sequence identities of the GPCR orthologue pairs is also lower than for the whole genomes. We found these to be 80% for the rat and human pairs and 90% for the rat and mouse pairs. However, the proportions of orthologous and species-specific genes vary significantly between the different GPCR families. The largest diversification is seen for GPCRs that respond to exogenous stimuli indicating that the variation in their repertoires reflects to a large extent the adaptation of the species to their environment. This report provides the first overall roadmap of the GPCR repertoire in rat and detailed comparisons with the mouse and human repertoires.

## Background

The rat genome [1] was the third mammalian genome to be sequenced, after the human [2,3] and mouse [4] genomes. The rat is one of the most widely used model organisms in biological research. Despite this fact, the annotated proteins are fewer for rat (26,123) compared to mouse (30,397) and human (41,973) (Ensembl "peptides known" and "peptides known-ccds" datasets March 2007) [5]. Automated protein predictions offer fast annotation but they are error-prone and need to be followed up by careful manual curation. For instance the Genscan gene prediction program has a sensitivity and specificity of about 90% for detecting exons, leading to frequent errors in multi-exon genes [6]. Our recent annotation of the chicken G protein-coupled receptors (GPCRs) showed that over 60% of the chicken Genscan gene predictions with a human orthologue needed curation. The curation drastically increased the quality of the dataset as the average percentage identity between the human-chicken one-to-one orthologous pairs was raised from 56% to 73% [7]. Moreover, automated annotation based on inter-species comparisons has difficulties in identifying species-specific proteins, a problem that is relevant especially to large protein families that display large differences between species. For example previous reports of the V1R and V2R vomeronasal receptor repertoires display a very large variation in numbers [8-11]. The degrees of completeness and accuracy of protein annotations have, of course, a substantial impact on subsequent analyses such as phylogenetic analyses and calculations of evolutionary distances. Accurate comparisons of the rat and human proteins, such as correct assignment of orthologous pairs, are crucial for the design and interpretation of physiological and pharmacological studies in which results are inferred between the species.

The superfamily of GPCRs is one of the largest groups of proteins within most mammals. GPCRs are signal mediators that have a prominent role in most major physiological processes at both the central and peripheral level [12]. It has been estimated that about 80% of all known hormones and neurotransmitters activate cellular signal transduction mechanisms via GPCRs [13]. The key common structural components of the GPCRs are the seven transmembrane α-helices that span the cell membrane. It has been estimated that GPCRs represent between 30–45% of the current drug targets [14,15]. However, drugs have only been developed for a very small number of the GPCRs and the potential for further drug discovery within this field is enormous.

The human GPCR repertoire has previously been divided into five main families (GRAFS); *Glutamate *(clan C), *Rhodopsin *(clan A, includes the olfactory receptors), *Adhesion *(clan B2), *Frizzled*/*Taste2 *and *Secretin *(clan B) [16]. The GRAFS families are found in all bilateral species and it has thus been suggested that they arose before the split of nematodes from the chordate lineage [17]. The largest family is by far the *Rhodopsin *family, one reason being that it includes the many olfactory receptors (ORs). Most of the GPCR drug targets, mainly amine and peptide receptors, are found within this family [18]. In humans, the second largest family is the *Adhesion *family. Most of the receptors in this family are still orphans (without a known ligand) [19,20]. The *Glutamate *family includes receptors that bind to glutamate, GABA and calcium as well as the groups of sweet and umami taste receptors (TAS1Rs) and vomeronasal receptors type 2 (V2Rs) that are binding native exogenous compounds. The *Secretins *bind large peptides such as secretin, parathyroid hormone, glucagon, glucagon-like peptide, calcitonin, vasoactive intestinal peptide, growth hormone releasing hormone and pituitary adenylyl cyclase activating protein. The *Frizzled *receptors bind among others the Wnt ligand and play an important role in embryonic development. The vomeronasal receptors type 1 (*Vomeronasal1) *and taste receptor type 2 (*Taste2) *GPCR families are involved in pheromone recognition and bitter taste sensing, respectively. Moreover, there are gene sequences that have been suggested to represent additional GPCRs but show only weak or no sequence similarity to any of the main GPCR families.

Here we provide the first overall map of the GPCR subset of the rat genome. We have made extensive efforts to mine the GPCR repertoire and cover all GPCR families comprising a total of 1867 (+739 pseudogenes) gene sequences. In addition we have also updated the human and the mouse repertoires. We have performed phylogenetic analyses of the rat, human and mouse GPCR families and provide detailed information of their orthologous relationships.

## Results

### The overall GPCR repertoires in rat, mouse and human

In this analysis we present the overall repertoire of GPCRs in rats and updated versions of the GPCR repertoires in humans and mice. The GPCR families found in humans, GRAFS, have been slightly updated since they were last described by our group, but the mouse datasets are nearly identical [21]. The main difference is that in this analysis the *Vomeronasal1 *family and the olfactory receptor (OR) group are included and the *Frizzled *and *Taste2 *receptors are considered as two separate families. Throughout this article the GPCR families are written in italics; *Adhesion*, *Frizzled*, *Glutamate*, *Rhodopsin*, *Secretin*, *Taste2 *and *Vomeronasal1*, whereas the names of family subgroups are abbreviated e.g. V2R (vomeronasal type 2 receptors) and OR (olfactory receptors). Our classification of the GPCR families is based strictly on sequence homology. This analysis also includes 21 gene sequences that have been suggested to encode GPCRs but do not belong to any of the GPCR families (i.e. they have no sequence similarity). These are referred to as *"Other GPCRs" *or just *"Others"*. The numbers of GPCR genes in the different families and species are given in Table 1 and the names of the receptors in each family and species is listed in a separate spreadsheet [see Additional File 1]. In Table 1, the GPCR genes have been categorised as "full-length", "pseudogenes" or "partial" and the numbers of new genes are also indicated. Full-length GPCR genes contain an intact transmembrane domain and are likely to encode functional receptor proteins. The pseudogenes are non-functional genes that have been frame-shifted due to additions or deletions of nucleotides or truncated by missense mutations introducing stop codons. The "partial" genes are missing parts of their sequence due to incomplete information in the respective genome assembly and in sequence databases. GPCR gene sequences were defined as new if they had not been annotated as GPCRs in Genbank and did not exist in any of the supplementary datasets linked to previous studies. The GPCR repertoires in the rodents are more than twice as large as that in humans, and rats have more GPCRs than mice. This is mainly explained by the large differences in the number of ORs, but a substantial part of the human-rodent differences is because of the vomeronasal receptors, *Vomeronasal1s *and V2Rs (a subset within the Glutamate family). There are no partial human GPCR genes, 2 in mice and 38 (1.4% of all genes) in rats. These figures reflect the completeness of the respective genome assemblies which must now all be considered fairly complete. Most (28/38) of the partial rat sequences are OR genes. These are difficult to assemble due to their large numbers, high sequence similarity and genomic proximity. In this respect this analysis shows an important difference as compared with for example the initial work on mouse by Vassilatis and colleagues [22], which contained large numbers of partial sequences. Our sequence datasets [see Additional files 2, 3, 4] have, in all instances possible, been carefully compared to previously published datasets (see below).

**Table 1:** The human, rat and mouse GPCR family repertoires

	**Human**					**Rat**						**Mouse**					
**GPCR Family**	Full- length	Pseudo- genes	Pseudo- genes (%)	New full- length	New pseudo- genes	Full- length	Pseudo- genes	Partial	Pseudo- genes (%)	New full- length	New pseudo- genes	Full- length	Pseudo- genes	Partial	Pseudo- genes (%)	New^1 ^full- length	New^1 ^pseudo- genes
			
*Adhesion*	33	2	6%	0	2	26	1	4	3%	0	2	30	1		3%	0	0
*Frizzled*	11	0	0%	0	0	10	1		9%	0	0	11	0		0%	0	0
*Glutamate*	22	0	0%	0	0	22	0		0%	0	0	22	0		0%	0	0
-V2R subgroup	1	11	92%	1	11	108	87	2	45%	23	53	111	183	1	62%	21	63
*Rhodopsin*	284	27	9%	0	1	297	7	4	2%	5	0	320	23		7%	3	NA*
-OR subgroup	388	479	55%	0	12	1234	552	28	31%	1234	552	1081	325	1	23%	NA*	NA*
*Secretin*	15	0	0%	0	0	15	0		0%	0	0	15	0		0%	0	0
*Taste2*	25	10	29%	0	0	35	6		15%	0	0	35	5		13%	0	0
*Vomeronasal1*	5	53	91%	0	53	105	84		44%	12	76	145	164		53%	17	92
*Other GPCRs"*	15	1	6%	0	1	15	1		14%	1	0	13	1		7%	1	0
Total	799	583		1	133	1867	739	38		1276	759	1783	702	2		43	247

The human, rat and mouse GPCR families; their numbers of full-length, pseudogene and partial members; as well as the number of new members identified in this study. GPCR gene sequences were defined as new if their sequence was not available in a public database annotated as a GPCR. *No figure could not be calculated.

### Phylogenetic analyses

**Figure 1:**
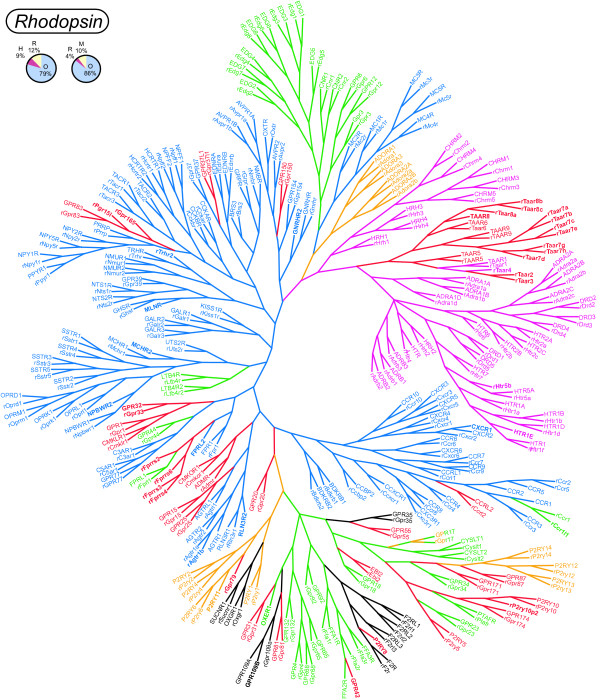
**Phylogenetic analysis of the *Rhodopsin *family**. The figure shows a phylogenetic tree of *Rhodopsin *family receptors. *Rhodopsins *which display ambiguous relationships were analysed in separate (see Figure 2). Species-specific receptors have names in bold style. The ligand types of the receptors are indicated with the following colours; red: orphan, blue: peptide, lilac: amine, green: lipid-like, brown: purine, turquoise: opsin and black: other. The first pie chart above the trees shows the proportions of human-rat one-to-one orthologues (O), human specific (H) and rat specific (R) members. The second pie chart displays the proportions of rat-mouse one-to-one orthologues (O), rat specific (R) and mouse specific (M) members. This phylogenetic tree is a consensus tree of 2 consensus trees derived from 100 maximum parsimony and neighbour joining analyses, respectively, and calculated using the UNIX version of the Phylip 3.6 package [73].

**Figure 2:**
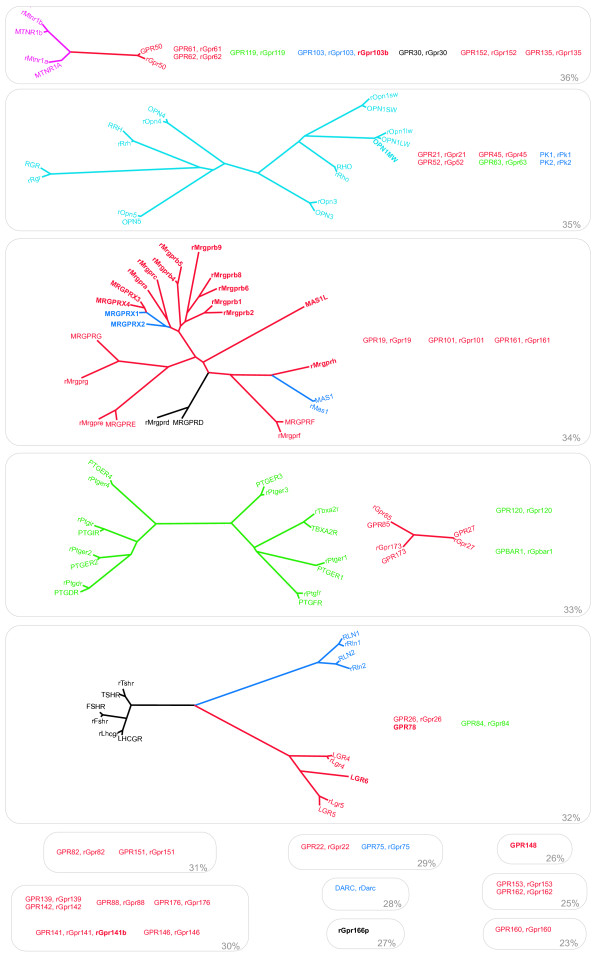
***Rhodopsin *family receptors with ambiguous relationships to other members**. Groups, pairs and "single" *Rhodopsin *family receptors that display ambiguous relationships to other members (see material and methods). These are shown in order of decreasing sequence identity (given in the lower right corner of each box). Species-specific receptors have names in bold style. The ligand types of the receptors are indicated with the following colours; red: orphan, blue: peptide, lilac: amine, green: lipid-like, brown: purine, turquoise: opsin and black: other. The phylogenetic trees are consensus trees of 100 maximum likelihood trees for which branch lengths were calculated using TreePuzzle (see material and method). The olfactory subfamily/group of the *Rhodopsin *family was analysed in a separate phylogenetic analysis [see Additional files 7 and 8].

**Figure 3:**
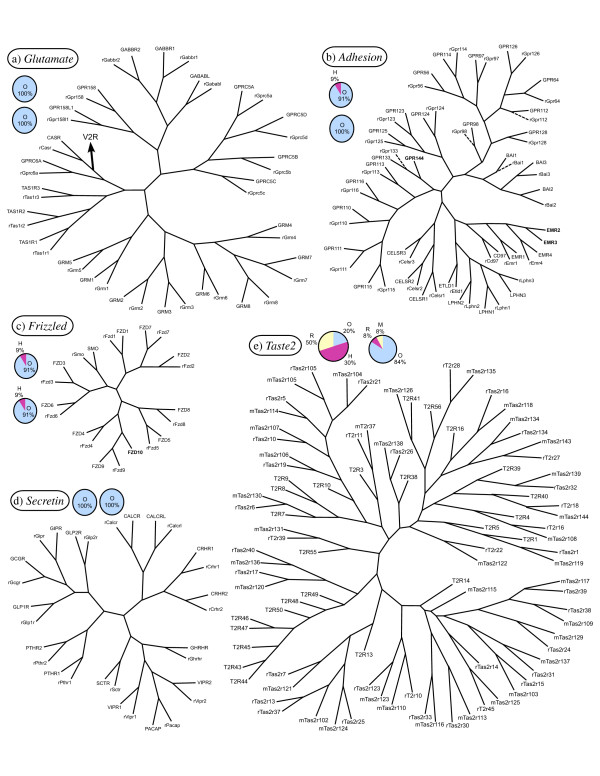
**Phylogenetic trees of the *Glutamate, Adhesion, Frizzled, Secretin *and *Taste2 *families**. The figure shows the consensus trees of 100 maximum parsimony trees of the human and rat a) *Glutamate*, b) *Adhesion*, c) *Frizzled*, d) *Secretin *and e) *Taste2 *(mouse included) GPCR families. The first pie chart after the each family name shows the proportions of human-rat one-to-one orthologues (O), human specific (H) and rat specific (R) members. The second pie chart displays the proportions of rat-mouse one-to-one orthologues (O), rat specific (R) and mouse specific (M) members. In Figure 3, the place of the large group of V2Rs is indicated with an arrow. Rat sequences that were excluded from the phylogenetic analysis because they were incomplete (due to gaps in the genome assembly) are displayed with dashed branches.

We produced phylogenetic trees for each GPCR family. These are shown in Figures 1, 2, 3 and as additional files of this article [see Additional files 5, 6, 7], which all also hold pie charts of the proportions of orthologous and species-specific GPCRs in the three species analysed. The mouse *Glutamate, Rhodopsin *(non-ORs), *Adhesion, Frizzled *and *Secretin *GPCRs are not included in these trees since they have been described before in relation to the human repertoires [21], and the exact orthologous and paralogous relationships are viewed in a table listing the receptors in the three species studied [see Additional File 1]. All receptors sequences were trimmed prior to the phylogenetic analysis to isolate the transmembrane domains which are the regions that are conserved throughout the GPCR families. A special approach was used for the phylogenetic analysis of the *Rhodopsin *family which is very challenging because of its size and diversity. Some *Rhodopsin *GPCRs have diverged to the extent that their relationships to the rest of the family members cannot be determined. These receptor sequences do not show stable tree topology and group with different subfamilies when using different phylogenetic algorithms. The ambiguous grouping of these receptors is also illustrated by the fact that when their sequences are searched against the whole dataset using BLAST the best matches (e.g. top 5) to these receptors belong to different subfamilies. We minimised the above problems by separating the receptors with a low sequence identity from the main part of the dataset when performing phylogenetic analyses. This was done by applying iteratively higher thresholds of percentage sequence identity on the dataset until a stable tree topology could be obtained.

### The *Rhodopsin *family

The phylogenetic tree(s) of the *Rhodopsin *family in human and rat is shown in Figure 1 and 2. The majority of the *Rhodopsins *are in Figure 1, whereas other members with ambiguous relationships are shown in Figure 2 as separate groups, pairs and singles in the order of falling sequence identity (to the sequences before them). Sequences defined by the same percentage of sequence identity are grouped in boxes with the threshold value given in the lower right corner. In Figure 1 the α-δgroups, previously defined by our group [16] were reproduced and they contain most of the receptors from the original analysis. 79% of the human and rat *Rhodopsins *are found in one-to-one orthologous pairs.

### *Rhodopsin *family species variation

Several of the *Rhodopsin *family GPCRs show interesting species variation. Most of the species-specific *Rhodopsins *are found among the MAS-related GPCRs (MRGs), trace-amine-associated receptors (TAARs) and "formyl peptide receptor-like" (FPRLs) receptors. The group of MRGs are located primarily in sensory neurons of the dorsal root ganglion and are implicated in perception of pain [23]. The numbers of MRGs (at the 34% threshold) are 10, 15 and 28 in human, rat and mouse, respectively. There are 5 rat-human orthologous pairs of MRG receptors. The TAARs (in the cluster of amine binding receptors) are 5, 15 and 17 in human, mouse and rat, respectively [24,25]. The FPRLs (slightly below and to the left of the centre of the largest tree) Fprrs2, Fprrs3, Fprrs4 and Fprrs6 are rat specific, whereas FPRL2 is only found in humans. The remaining species-specific *Rhodopsins *are singles (shown in bold style in Figure 1, 2) derived from a single gene duplication or gene loss in either the primate or rodent lineage. These are: 5-HT1E and 5-HT5B serotonin receptors (HTR1E and Htr5b; among the amine binding receptors); Gonadotropin releasing hormone receptor 2 (GnRHR2), Gpr165, Pgr15l, Thyrotropin releasing hormone receptor 2 (Trhr2) and the motilin receptor (MLNR) (among the many peptide receptors); MCHR2 and NPBWR2 (at the roots of the somatostatin and opioid receptors, respectively); GPR32 and Gpr33 (under the somatostatin and opioid receptor cluster in Figure 2); Agtr1b and RLN3R2 (left of the chemokine and purine receptor clusters); P2RY11, Gpr79, OXER1, GPR109B, GPR42, P2RY8, P2ry10p2 (among the purine and F2R receptor cluster); CXCR1, and Ccr1l1 (among the chemokine receptors); Gpr103b (36% threshold); OPN1MW (35% threshold); GPR78, LGR6 (32% threshold); Gpr141b (30% threshold); Gpr166p (27%.) and GPR148 (26% threshold).

### *Rhodopsin *family ligand types and orphan receptors

The colour coding of Figure 1 and 2 gives an overview of the types of ligands or bound proteins for the *Rhodopsin *family receptors. There are 100 peptide (blue), 45 lipid (green), 38 amine (lilac), 13 purine (brown) -binding human *Rhodopsins *and also 9 opsin receptors that are by light (turquoise). One receptor, GPR17 has a dual ligand specificity for purine and lipid molecules [26]. The remaining 14 *Rhodopsins *(black) are scattered in smaller groups of ligand types including protease-activated (F2R, F2RL1, F2RL2 and mF2RL3), glycoprotein hormone (FSHR, LHCGR and TSHR), metabolite (OXGR1, GPR35, SUCNR1, GPR109A and GPR109B), amino acid (MRGPRD) and steroid hormone (estrogen receptor GPR30). Moreover, we found that there are 65 human orphan *Rhodopsins*, of which 56 have orthologues in rats. 27 of the human orphan *Rhodopsins *are grouped in the phylogenetic tree of the majority of the receptors (Figure 1), whereas 38 have more atypical sequence and are found in smaller groups, pairs or are singles (Figure 2). 27 of the human orphan *Rhodopsins *could not be grouped with any characterized receptor making ligand prediction difficult. Additional information about the phylogenetic grouping of ligand types and orphan receptors was presented in a recent study by Surgand et al [27].

### The *Glutamate, Adhesion, Frizzled, Secretin *and *Taste2 *families

The *Glutamate *(except for the V2R group) (Fig. 2a) and *Secretin *(Fig. 2d) families have a completely conserved number of members between rats, mice and human and they are all one-to-one orthologues. GPCRs belonging to the Glutamate family have been shown to form functional heterodimers or homodimers. The GABAB receptor is formed by heterodimerization between one GABAB1 subunit and one GABAB2 subunit, the sweet taste receptor is formed by heterodimerization between the T1R2 subunit and the T1R3 subunit, whereas the umami taste receptor is formed by heterodimerization between the T1R1 subunit and the T1R3 subunit. Additional GPCRs within the Secretin family are formed by heterodimerization between a GPCR and an accessory protein [28]. In the *Frizzled *family (Fig. 2c) one rat member, Fzd10, is a pseudogene. Two *Adhesions *(Fig. 2b), EMR2 and EMR3, were previously shown to be missing in mice [29] and these are also missing in the rat genome. Moreover, we now found that the rat and mouse gene sequences of Gpr144 contain stop codons within the transmembrane region and are thus pseudogenes. We recently reported on two new human pseudogenes of the *Adhesion *family, both similar to GPR116 [21] and these have now received official names from HUGOs Gene Nomenclature Committee and are now referred to as GPR116P1 and GPR116P2, respectively. The rodent and human *Taste2 *families (Fig. 2e) have a low proportion of one-to-one orthologous pairs. Among the 35 rat, 35 mouse and 25 human receptors, there are only 10 orthologous triplets (one copy from each species). These represent 20% of all members, whereas in rats and mice, 91% of the *Taste2s *make up one-to-one orthologous pairs. The number of *Taste2s *in our datasets is equal or close to previously reported numbers [30-35]. The rat receptors T2R39 and T2R45 [33] are not available in any public sequence database.

### *The Vomeronasal1 *receptor family

Five full-length *Vomeronasal1 *genes, VN1R1-5, have been previously identified from sequence mining in the human genome assembly [8]. We removed VN1R3 from this list because it contains a frame shift in the current genome assembly, but we found a new intact human *Vomeronasal1*, FKSG83. The number of human *Vomeronasal1 *pseudogenes was first estimated at 195 [8], but this number was later reduced to 115 [9] and in this study we found only 53. We found 145 full-length genes and 164 *Vomeronasal1 *pseudogenes in the mouse genome and 105 full-length genes and 84 pseudogenes in the rat genome. These numbers are within the ranges of previously reported figures which are 137–187 full-length and 156–168 pseudogenes for mouse and 95–106 full-length and 21–110 pseudogenes for rat [9-11,36,37]. We found many non-*Vomeronasal1s *and duplicates within the datasets from Young *et. al*. [9]. A phylogenetic tree of the *Vomeronasal1 *family was produced [see Additional File 5]. Only 9% of the rat-mouse receptors are one-to-one orthologues and there are no human-rat one-to-one orthologues.

### The vomeronasal type 2 receptors (V2Rs)

The human V2R repertoire has not been mined in our previous whole genome GPCR repertoire articles and here we identified 1 full-length gene and 11 pseudogenes. Moreover, we almost doubled the number of reported intact V2Rs in rat (from 61 to 108) and mouse (from 57 to 111) and raised the total numbers of receptors considerably (168 to 197 in rat and from 209 to 295 in mouse) [38]. Our phylogenetic tree of the V2Rs is available as an additional file [see Additional File 6]. There are no human-rat orthologues in this receptor group and only 2% of the rodent V2Rs are orthologues.

### The olfactory receptors (ORs)

The olfactory receptors make up half or more of all GPCRs in mammalian species (human: 49%, mouse 61% and rat 66%) and they constitute 1,7%, 4,5% and 5,3% of the genes in the human, mouse and rat genomes, respectively (gene numbers from Ensembl [39]). Our search for human ORs identified all (388) of the full-length genes and 96% of the pseudogenes in the HORDE database [40], and in addition we found 12 new pseudogenes. This shows that the sensitivity and specificity of our searches was high. We identified 1081 full-length OR genes and 325 OR pseudogenes in the mouse genome which is close to what was reported in a recent analysis which resulted in 1037 full-length genes and 354 pseudogenes [41]. We found 1814 ORs (1234 full-length genes, 552 pseudogenes and 28 partial genes) in the rat genome. The rat therefore has at least 153 (14%) more full-length OR genes and 227 (70%) more OR pseudogenes than mice and 846 (218%) more full-length OR genes and 73 (15%) more OR pseudogenes than humans. The percentages of pseudogenes are 23% in mouse, 31% in rats and 55% in humans. An OR phylogenetic tree with pie charts of the proportions of orthologues and the large tree file in Newick format were produced [see Additional files 7 and 8]. 8% (120) of the rat and human ORs are orthologous, 18% (268) are human specific and 74% (1115) are rat specific. Of the mouse and rat ORs 31% (550) are orthologous, 30% (534) are mouse specific and 38% (675) are rat specific.

#### *"Other GPCRs"*

The additional sequences suggested by various sources to be GPCRs, here called *"Other GPCRs" *or just *"Others"*, are shown in Table 2. There are 4 pairs (GPR107–GPR108, GPR177–GPR178, GPR172A-GPR172B, TMEM185A-Tmem185a) and 2 triplets (GPR137-GPR137B-GPR137C, PAQR5-PAQR7-PAQR8) of homologues within this set. No additional sequences related to these have been found in the human, mouse and rat genomes except for the PAQRs that have additional homologues, but these are not suggested to be GPCRs [42]. Thus the "*Others" *(except the PAQRs) seem to make up protein families of their own comprising only 1–3 members. GPR143 (OA1) is the only one in this list that has been shown to be able to activate a G protein [43]. For PAQR5 different reports describe either plasma membrane or intracellular location [44,45]. PAQR5 GPR149 (IEDA), Gpr181p (pseudogene in human) and GPR157 have vague and ambiguous sequence similarities to several different GPCRs families. GPR107, GPR108, GPR137, GPR137B and GPR137C show low similarity to various membrane bound proteins. Only GPR143, GPR149, Gpr181p and GPR157 show a higher similarity to membrane proteins than other proteins and 10 *Others *are indicated to have more or less than 7 transmembrane regions. Some sequences suggested to be GPCRs have later been shown to be members of other membrane protein families. We found one such example when searching in the non-redundant database for alternative versions of putative GPCR sequences. A protein [GenBank: BAB15283.1] recently discovered by searches utilising Hidden Markov Models and suggested to be a GPCR [46] is a sugar transporter, MYL5/MFSD7 [GenBank: AAQ88767] comprising 12 transmembrane helices. Two additional human GPCR sequences from the same study have now received GPR names, GPR177 and GPR178, by HGNC upon our request and are found among the *Others *in Table 2.

**Table 2:** "Other GPCRs"

**GPCR (alias)**	**#TM helices**	**Conserved domains (e-value)**	**First BLAST hits (best e-value)**
PAQR5	6	HlyIII (3e-23)	PAQR8 (6e-26), PAQR7 (6e-24), other PAQRs
PAQR7	6	HlyIII (2e-20)	PAQR8 (2e-73), PAQR5 (1e-25), other PAQRs
PAQR8	6	HlyIII (4e-27)	PAQR7 (6e-24), PAQR5 (1e-26), other PAQRs
GPR143 (OA1)	6	7TM_2 (3e-3), 7TM_1 (0.017)	Secretin (0.09), Adhesion (0.091)
GPR149 (IEDA)	7	7TM_1 (1e-6)	Rhodopsin (0.5)
Gpr181p	7	7TM_1 (2e-3)	V1R (3e-5), Rhodopsin (3e-4), TAS2R (9e-3)
GPR157	7	7TM_2 (6e-13), 7TM_1 (8e-6), FZD (2e-4)	Secretin (1e-4), Rhodopsin (1e-4), Adhesion (9e-4)
GPR107	7	Lung_7-TM_R (9e-76)	GPR108 (2e-130), membrane proteins (1e-6)
GPR108	7	Lung_7-TM_R (9e-78)	GPR107 (e-132), membrane proteins (4e-8)
GPR137 (C11ORF4)	7	-	GPR137B (9e-75), membrane proteins (7e-16)
GPR137B (TM7SF1)	7	-	GPR137 (6e-99), membrane proteins (1e-6)
GPR137C (TM7SF1L2)	7	-	GPR137B (3e-60), different proteins (1e-4)
TM7SF3	7	-	-
TM7SF4 (FIND, DC-STAMP)	6	-	different proteins (3e-9)
GPR175	7	-	-
GPR177	8	DUF1171 (4e-130)	GPR178 (6e-5)
GPR178	9	MARCKS (1e-3), STOP (5e-3), APC (7e-3), IER (8e-3)	GPR177 (1e-5)
TMEM185A	8	-	Tmem185b (4e-169), uncharacterised proteins
Tmem185b	7	-	TMEM185A (7e-169), uncharacterised proteins
GPR172A (PERVAR1)	11	DUF1011 (3e-29)	GPR172B (4e-136), uncharacterised proteins
GPR172B (PERVAR2)	11	DUF1011 (3e-32)	GPR172A (1e-144), uncharacterised proteins

*"Other GPCRs"*; sequences that have been suggested to be GPCRs but are not members of any GPCR family. The searches for transmembrane helices were made using TMHMM version 2.0 [86]. Conserved domains were searched for using conserved domain search [71] with a cut-off at e = 0.01. The BLAST searches for the most similar proteins were performed in the Refseq database using a cut-off at e = 0.01.

## Discussion

Here we present the first overall analysis of the GPCR subset of the rat genome and we provide updated versions of the human and mouse GPCR repertoires. 1276 (68%) of the full-length rat GPCRs had not been previously annotated and we also identified 40 new mouse receptors and 1 new human receptor. Of the new receptors in this study, 93% are ORs and 6% are vomeronasal receptors. We have performed comprehensive sequence mining and our datasets have been carefully compared with previously available versions. The GPCR gene sequences have been verified for a complete and non-interrupted coding region and incorrectly predicted sequences have been manually curated. We also performed phylogenetic analyses displaying for the first time the detailed orthologous relationships of the GPCR repertoires in rats, mice and humans. This information is valuable when results from pharmacological and physiological studies are compared or extrapolated between species.

The authors of the draft rat genome assembly estimated the proportion of one-to-one orthologues to be 89–90% between the rat and human genomes and 86–94% between the rat and mouse genomes [1]. The average amino acid sequence identities of the orthologues were reported to be 88.3% for rat and human and 95.0% for rat and mouse. Remarkably, we find that the average proportion of one-to-one orthologues within the GPCR superfamily is much lower. The average proportions of such orthologues were only 58% for the rat and human, and 70% for the rat and mouse GPCR repertoires. The average protein sequence identities of the GPCR orthologue pairs is also lower than for the whole genomes. We found these to be 80% for the rat and human pairs and 90% for the rat and mouse pairs. This suggests that the GPCR superfamily is much more divergent than the protein families in the genome in general. However, this difference in the proportions and sequence identities of orthologues may not entirely be explained by the relatively high divergence of the GPCR superfamily. This is also related to the fact that we have mined and edited the sequences to a much more complete level than the sequence data from the original draft rat genome assembly.

There are very different levels of evolutionary conservation or orthologous pairing of the GPCR families. There are families in which all (*Glutamate *(except the V2R group) and *Secretin *families) or almost all (*Adhesion *and *Frizzled) *receptors are conserved between the compared species. Other families have expanded separately in the three species (primarily the *Taste2s*) or expanded in one lineage while vanishing (*Vomeronsal1 *and V2R) in another. There are also remarkable examples of family subgroups that have all expanded in the rodents while diminishing in primates (olfactory receptors (ORs), trace-amine-associated receptors (TAARs), MAS-related GPCRs (MRGs), formyl peptide receptor like receptors (FPRLs) and vomeronasal type 2 receptors (V2Rs)). It is notable that all the large inter-species variations are found among the families and subgroups that respond to exogenous stimuli. Thus these differences are likely to make important contributions to the diversification of the senses of olfaction, taste and pheromone sensation among the species. The pheromone sensation has evolved in rats and mice, which have many species-specific vomeronasal receptors (*Vomeronasal1s *and V2Rs), but disappeared in the primate lineage in which the vomeronasal organ is regressed in adults and critical components of vomeronasal transduction pathway have been lost [47,48]. Moreover, it has recently been shown that the TAARs may act as chemosensory receptors in the olfactory epithelium [49,50].

There is a considerable variation among the different families and groups of GPCRs in the percentage of amino acid identity of orthologues. The rat and human *Frizzled *family orthologues have 95% average sequence identity while the lowest percentage identity between rats and human orthologous pairs is found in the *Taste2 *family (58%). The *Taste2 *family also display a very high divergence with respect to the repertoire. Only 20% of the rat and human and 84% of the rat and mouse receptors are one-to-one orthologues, indicating that this family is evolving rapidly. The *Taste2 *receptors mediate the sense of bitter taste [51] and it is likely that their divergence has contributed to the different capacities of species to recognise bitter tastants. Also the *Adhesion *family GPCRs display relatively low amino acid identity between rat, mouse and human orthologues (72%). However, in contrast to the *Taste2 *family, the *Adhesion *family repertoire is relatively well conserved; 100% of the rat and mouse and 91% of the rat and human *Adhesions *make up one-to-one orthologous pairs. The fact that the *Adhesions *have been retained during evolution shows that they have important physiological functions and their relatively low sequence conservation indicate that their action is less dependent on the transmembrane region. This is in agreement with the hypothesis that the transmembrane regions of the *Adhesion *GPCRs may perhaps not be involved in direct interactions with ligands or G-proteins but have an important role as membrane anchors [52].

The human ORs have previously been divided into class I and class II based on phylogenetic criteria [53]. The human genome contains 53 (14%) class I and 335 (86%) class II ORs and our analysis shows that the rat genome includes 139 (11%) class I ORs and 1096 (89%) class II. Class I ORs mediate the effect of water-soluble odorants and class II receptors recognise airborne odorants [53,54]. The proportion of rat and mouse OR orthologues is relatively low (31%) and the proportion of rat and human OR orthologues is very low (8%). Species-specific receptors can be found in most parts of the phylogenetic tree, but there are also several large clusters of species-specific ORs [see Additional File 8]. There are five large clusters containing exclusively rat-specific ORs. These are located closest to the human OR1F (18 rat ORs), OR1I1 (7 rat ORs), OR7A5,10,17 (6 rat ORs), OR2AK2 (6 rat ORs), OR2B11 (9 rat ORs) (for nomenclature, see [53]). Many human-specific ORs were found in the OR2T-group which contains 13 human ORs (OR2T1–7, 10–11, 27, 29, 34, 35) without a rodent orthologue. These species-specific clusters include receptors exclusively belonging to class II suggesting that this differentiation between the species has contributed to the unique capacities to recognise volatile airborne substances.

It is difficult to envision why in some cases several species lack a certain receptor while the same receptor has been retained during evolution in other lineages/species. One of the most likely explanations is that the gene has been lost because another gene has taken over its functions. One example of this could be the motilin receptor, MLNR. We did not find orthologues to this human receptor in neither rat nor mouse. In further searches (data not shown) we did however, find orthologues for this receptor in chimps (partial sequence), cow, chicken, zebrafish and pufferfish (Tetraodon nigroviridis) but not in dogs, suggesting that this receptor has been specifically lost in several mammals. MLNR is most closest related to the ghrelin receptor, GHSR (named after the subfragment of the growth hormone secretagogue peptide) and these receptors have 44.2% overall protein sequence identity. Motilin stimulates gastrointestinal motility and this function is modulated by a pharmaceutical substance, erythromycin A, which mimics this peptide and targets the MLNR receptor [55,56]. It has been shown that the ghrelin peptide with its receptor regulates gastrointestinal motility in rodents and the expression of GHSR in the human gastrointestinal tract indicates that ghrelin plays a role in gastrointestinal motility in humans as well [57-59]. It is thus possible that the ghrelin system plays a compensating role in GI motility in species that are missing the motilin receptor, such as rodents and dogs.

There are several additional cases in which two *Rhodopsin *family receptors have the same ligand and could share functions in some species while one of the receptors has been lost in several other evolutionary lineages such that the other gene may have taken over. TRHR and Trhr2 both bind to thyrotropin-releasing hormone (TRH). The former receptor has been conserved throughout all searched vertebrate genomes whereas Trhr2 is lacking in primates, chicken and fugu suggesting that it has been lost independently in three lineages. Furthermore the neuropeptide B/W receptors (NPBWR1 and NPBWR2) have a 62.2% overall sequence identity in human. We found both NPBWR1 and NPBWR2 in fish but whereas NPBWR1 has been retained in the examined vertebrate species NPBWR2 is found neither in the dog nor the chicken genomes and has become a pseudogene in the rodent lineage. Another similar case is seen for the melanin-concentrating hormone receptors MCHR1 and MCHR2. We could find both receptor subtypes in fish but whereas the former has been conserved the latter, MCHR2, is absent or is a pseudogene in rat, mouse, hamster, guinea pig and chicken [60]. This is illustrated also for another *Rhodopsin *family receptor, OXER1, which is missing in the rodents and in chicken but is conserved in fish, primates, cow and dog (1 full-length gene and 1 pseudogene). For this receptor there exists no characterized homologue that binds the same ligand (5-oxo-ETE) but there are several closely related orphan receptors such as GPR31, GPR81, GPR109A and GPR109B (primate specific duplicate) that could tentatively be activated by the same ligand.

In summary, we have presented the overall GPCR repertoire of rats and analysed it in relation to updated datasets for human and mouse. We have identified new genes, made extensive comparisons to previously published datasets and performed phylogenetic analyses to distinguish orthologous and species-specific receptors. This material is important for the interpretation of studies in which results are extrapolated between species to provide information about receptor structure or native ligand.

## Conclusion

We present the first overall analysis of the GPCR repertoire in rats and compare this to versions of those in mouse and human. The receptor sequences have been manually curated to assure a higher level of completeness and quality. The detailed relationships of the repertoires, here determined by careful phylogenetic analyses, are valuable information when results from pharmacological and physiological studies are compared or extrapolated between species. The greatest diversification, with regard to both receptor numbers and sequences, is seen for those GPCRs that respond to exogenous stimuli indicating that the alteration of the repertoires of these receptors has been important for adaptation of the species to their environment. Our detailed comparison of GPCR repertoires also revealed several examples of species-specific expansions and deletions. We performed an extended analysis in additional species of several *Rhodopsins *that were shown to have been lost in different lineages but retained in others, investigating if their function might be shared with another (homologous) receptor. Most *Rhodopsin *family GPCRs that bind the same type of ligands, e.g. lipids and peptides, formed coherent groups in our phylogenetic analysis. However, many (28) of the human orphan *Rhodopsins *could not be grouped with any characterized receptor because of low sequence identity and/or ambiguous relationships making the prediction of the ligand difficult. We found the number of human orphan *Rhodopsin *receptors to be 65 while the number was 84 in 2002 [61] suggesting that the current rate of de-orphanization is higher than that for the discovery of members of this family.

## Methods

### Identification of rat GPCRs of the *Glutamate, Rhodopsin, Adhesion, Frizzled *and *Secretin *family receptors

The mouse GPCRs of the *Glutamate *(except V2R group), *Rhodopsin *(except OR group), *Adhesion, Frizzled *and *Secretin *families [21] were used as baits in stand alone BLASTP and TBLASTN searches [62] against NCBI non-redundant (nr) database [63]. For each and every query the accession numbers of the 20 first hits were collected and a non-redundant list was obtained which was used to collect the sequences of the hits. Sequence duplicates with different names were identified using BLASTCLUST [62] with the threshold set at 99% sequence identity. Remaining duplicates were splice variants or contained sequence polymorphisms or sequencing errors exceeding a base difference of 1% of the total sequence. These were identified from their genomic overlap as determined using stand alone BLAT against the June 2003 rat genome assembly. Missing rat orthologues were searched for using online BLAT [64] and TBLASTN against the nr and Celera databases [65].

### Identification of the human, rat and mouse OR, *Taste2*, *Vomeronasal1 *and V2R receptors

#### TBLASTN searches in genome assemblies

Query datasets were retrieved by downloading a start set of human, rat and mouse GPCR protein sequences from the Genbank database using the Entrez data-retrieval tool [66] in keyword searches. The downloaded datasets were searched using TBLASTN against the October 2005 releases of the Ensembl human (build 35), mouse (build 34) and rat (assembly 3.4) genome assemblies [5]. For the OR, *Taste2 *and *Vomeronasal1 *receptors, which are all intronless, we used the whole protein sequences. For the V2R, which have several exons, we used only the transmembrane region which is located within one exon. The cut offs used were e = 0.01 for the OR and rat V2R searches and e = 1 for the *Taste2 *and *Vomeronasal1 *searches.

#### Extracting gene sequences and removing non-GPCRs

The TBLASTN searches result lists, previously saved in tabular format, were processed so that overlapping hits on the same strand were merged using a custom made Java program (available upon request). The chromosome coordinates of the merged hits were used to extract the corresponding sequences from the genome using fastacmd of the NCBI blast package. Each sequence was elongated upstream until the first start codon and downstream until the first stop codon using a custom made Java program (available upon request). The preliminary datasets were "cleaned" from non-GPCRs and GPCRs from other families by searching it against the Refseq database using BLASTN with the default settings. The criterion used when including new GPCRs was that at least the first two hits had to belong to the same GPCR family. Protein translations were obtained using transeq from the EMBOSS package [67] and from these the longest intact coding domain was extracted. Protein translations were considered full-length proteins if they contained an intact seven transmembrane region. Proteins containing 5 or more unknown positions due to incompleteness in the genome assemblies were removed and put into a "partial" category of gene sequences.

### Naming the GPCRs

The human receptors have been named using the official Gene name and rat and mouse orthologues have been named after their human counterpart. For those human GPCRs which lacked official Gene names we requested new names from the HUGO Gene Nomenclature Committee (HGNC) [68]. We obtained new names for GPR177, GPR178 and GPR181P. We also received the official names for GPR116P1, GPR116P2, GPR166P and P2RY10P2 which had been previously been named by HGNC. The human ORs were named according to a widely accepted nomenclature (e.g. OR1A2) after matching our dataset to that of the HORDE database [40]. The *Vomeronasal1s *were named after existing nomenclatures for human [8], rat [11] and mouse [37,69]. There existed a nomenclature for the rodent V2Rs [38], which we used. For the *Taste2s *we used the official Gene names which are T2R-X in human and Tas2r-X in mouse, where X is a number. In rat these two nomenclatures are mixed and several members lack a Gene name. Other new receptors were assigned labels according to their chromosome coordinates prefixed by a letter indicating the species: human (H), rat (r) and mouse (m).

### Phylogenetic analyses

#### Removing non-conserved sequence regions

For the *Rhodopsins *the N-termini, C-termini and loops were defined from alignments to the protein sequence of the crystallised bovine Rhodopsin structure [70] and removed. For the other families NCBI's conserved domain search [71] was used to identify the borders of the transmembrane region and removing the N- and C-termini. The ORs and *Vomeronasal1s *were cut according to the "7tm_1" domain (*Rhodopsin *family), the *Taste2s *to the TAS2R domain, the *Glutamate *receptors (including the V2Rs) to the "7tm_3" domain (metabotropic glutamate family) and the *Adhesions *and *Secretins *to the "7tm_2" domain (Secretin family).

#### Division of the *Rhodopsin *family dataset

BLASTCLUST was used to apply a minimum threshold of protein sequence identity on a dataset consisting of all human *Rhodopsins*. The receptor with the lowest identity (less than 23%) to other *Rhodopsins *is GPR160 and this receptor was the first to be separated from the remaining dataset (Fig. 2). The clustering and separation procedure was repeated at increasing thresholds of sequence identity allowing the iterative separation of groups, pairs and single members with atypical sequences. This division of the dataset continued until we obtained reproducible topology of the human *Rhodopsins *in neighbor joining, maximum parsimony and maximum likelihood analyses (data not shown). This was achieved after the 36% threshold for sequence identity. All sequences that were separated from the main dataset were subject to BLAST searches against the whole human dataset. Queried/searched sequences which had all first five BLAST hits in the same cluster of related sequences were considered distant relatives and were merged with that particular cluster/dataset. The rat *Rhodopsin *receptors were merged with the corresponding dataset of human orthologues. A length threshold of 50% was used for the BLASTCLUST clustering.

#### Sequence aligning and bootstrapping

All datasets were aligned using CLUSTALW 1.82 [72] with default alignment parameters. Pseudogenes were excluded and the transmembrane regions extracted as described above. The stability of the phylogenetic branching was determined using bootstrap analyses. The alignments were bootstrapped 100 times using SEQBOOT from the UNIX version of the PHYLIP 3.6 package [73], except for the olfactory receptor (OR) alignment which was bootstrapped 10 times. This resulted in a total of 100 (10 for the ORs) different alignments from the original dataset, respectively.

#### Calculating phylogenetic trees

Unless otherwise specified the bootstrapped files were used for calculating maximum parsimony (MP) trees with PROTPARS from the PHYLIP 3.6 package. The trees were un-rooted and calculated using ordinary parsimony and the topologies were obtained using the built-in tree search procedure. From the bootstrapped OR alignment files protein distances were calculated using PROTDIST from the Win32 version of the PHYLIP 3.6 package. The Jones-Taylor-Thornton matrix was used for the calculation. The trees were calculated from the 10 different distance matrices, previously generated with PROTDIST, using NEIGHBOR from the same package. For the *Rhodopsin *family the largest (non-OR) group (Figure 2) was analysed with both MP and neighbor joining (NJ), whereas maximum likelihood (ML) was used for all other groups (Figure 3). For the latter groups branch lengths were calculated using the ML consensus tree as a user defined tree in TreePuzzle [74]. The following parameters were used; Type of analysis: Tree reconstruction; Tree-search procedure: User-defined trees; Compute clocklike branch lengths: Yes; Location of root: Best Place (automatic search); Parameter estimates: Exact (slow); Parameter-estimation uses: 1st input tree; Type of sequence input data: Amino acids; Model of substitution: VT Mueller-Vingron Model of Substitution, 2000); Amino acid frequencies: Estimate from dataset; Model of rate heterogeneity: Mixed (one invariable plus eight Gamma rates); Fraction of invariable sites: Estimate from dataset; Gamma distribution parameter alpha: Estimate from dataset; Number of Gamma rate categories: eight.

#### Merging and viewing the trees

The tree files were merged using the GNU-UNIX cat command and the resulting files were analysed using CONSENSE, from the PHYLIP 3.6 package, to retrieve bootstrapped consensus trees. The largest human-rat *Rhodopsin *family tree is a consensus tree of two other consensus trees, one from the NJ analysis and the other from the MP analysis. The trees were plotted using TREEVIEW and manually edited in CANVAS.

### Ligand type classification for the human *Rhodopsins*

An in-house list of ligand types for the human *Rhodopsins *was updated by obtaining missing ligand information from the receptor list maintained by the International Union of Pharmacology Committee on Receptor Nomenclature and Drug Classification (NC-IUPHAR) [75]. Receptors that did not have a ligand in this list were considered candidate orphan receptors. These were systematically searched using keyword look-ups in Pubmed and BLAST in the NCBI's non-redundant (nr) database to find receptor deorphanization reports [26,76-85]. The same GPCR often has several names and in order to obtain a higher coverage of publications we used names found from HGNC, NCBI Gene and the identical BLAST hits.

## Abbreviations

GPCR, G protein-coupled receptor; *Taste2*, taste receptor type 2 family of GPCRs; *Vomeronasal1*, vomeronasal receptor type 1 family of GPCRs; *Others *Other GPCRs; OR, olfactory receptor; V2R, vomeronasal type 2 receptor; HGNC, HUGO Gene Nomenclature Committee

## Competing interests

The author(s) declares that there are no competing interests.

## Authors' contributions

DEG carried out the receptor identification, performed the phylogenetic analyses and drafted the manuscript. RF participated in the design of the study and helped to draft the manuscript. HBS conceived the study, participated in its design and coordination and helped to draft the manuscript. All authors read and approved the final manuscript.

## Supplementary Material

Additional file 1 Comparative table of the *GRAFS(T) *family receptors in human, rat and mouse. A table listing the rat, mouse and human GPCRs of the *Glutamate, Rhodopsin, Adhesion, Frizzled, Secretin *and *Taste2 *families. Pseudogenes are marked with a "P" in the column to the right of the respective species. When several species- or lineage-specific duplicates (paralogues) exist the paralogue with the highest sequence identity has been given as the primary orthologue whereas the other genes are present on separate rows in the table and without a counterpart in the other species. Click here for file

Additional file 2 Rat GPCR gene sequences. The nucleotide sequences of all rat GPCRs compiled in this analysis, including pseudogenes. Note that this is a large file which will cover hundreds of pages if printed. Click here for file

Additional file 3 Human GPCR gene sequences. The nucleotide sequences of all human GPCRs compiled in this analysis, including pseudogenes. Note that this is a large file which will cover hundreds of pages if printed. Click here for file

Additional file 4 Mouse GPCR gene sequences. The nucleotide sequences of all mouse GPCRs compiled in this analysis, including pseudogenes. Note that this is a large file which will cover hundreds of pages if printed. Click here for file

Additional file 5 Phylogenetic tree of the *Vomeronasal1 *family. The figure shows the consensus trees of 100 maximum parsimony trees of the human and rat *Vomeronasal1 *GPCR family. The first pie chart above the tree shows the proportions of human-rat one-to-one orthologues (O), human specific (H) and rat specific (R) members. The second pie chart displays the proportions of rat-mouse one-to-one orthologues (O), rat specific (R) and mouse specific (M) members. Click here for file

Additional file 6 Phylogenetic tree of the vomeronasal type 2 receptors (V2Rs). The figure shows the consensus trees of 100 maximum parsimony trees of the human and rat vomeronasal type 2 receptors (V2Rs). The first pie chart above the tree shows the proportions of human-rat one-to-one orthologues (O), human specific (H) and rat specific (R) members. The second pie chart from the top displays the proportions of rat-mouse one-to-one orthologues (O), rat specific (R) and mouse specific (M) members. Click here for file

Additional file 7 Phylogenetic tree of the olfactory receptors (ORs). The figure shows the consensus tree of 10 neighbor joining trees of the human, rat and mouse olfactory receptors. The pie charts to the left show the proportions of human-rat one-to-one orthologues (O), human specific (H) and rat specific (R) members. The pie charts to the right display the proportions of rat-mouse one-to-one orthologues (O), rat specific (R) and mouse specific (M) members. The pie charts on the top give the proportions of one-to-one orthologues for all ORs, whereas the charts below contain the same information for the Class I and Class II subsets, respectively. Click here for file

Additional file 8 Phylogenetice tree file of the olfactory receptors (ORs). A Newick format consensus tree file from 10 neighbor joining trees of the human, rat and mouse olfactory receptors. Note that this is a very large tree file which can be difficult to open with many tree viewing programs and will cover a very large number of pages if printed. The file can be viewed in most tree viewing programs for example TREEVIEW which is available at no cost. Click here for file
