# Supplementary figures and images for: The G protein-coupled receptor subset of the rat genome
Source: BMC Genomics. 2007 Sep 25;8:338. doi: 10.1186/1471-2164-8-338 (PMC2117022; doi:10.1186/1471-2164-8-338)

*Vomeronasal1*

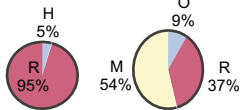

Supplement: Additional file 5 — Phylogenetic tree of the Vomeronasal1 family. The figure shows the consensus trees of 100 maximum parsimony trees of the human and rat Vomeronasal1 GPCR family. The first pie chart above the tree shows the proportions of human-rat one-to-one orthologues (O), human specific (H) and rat specific (R) members. The second pie chart displays the proportions of rat-mouse one-to-one orthologues (O), rat specific (R) and mouse specific (M) members. [file 1471-2164-8-338-S5.pdf]

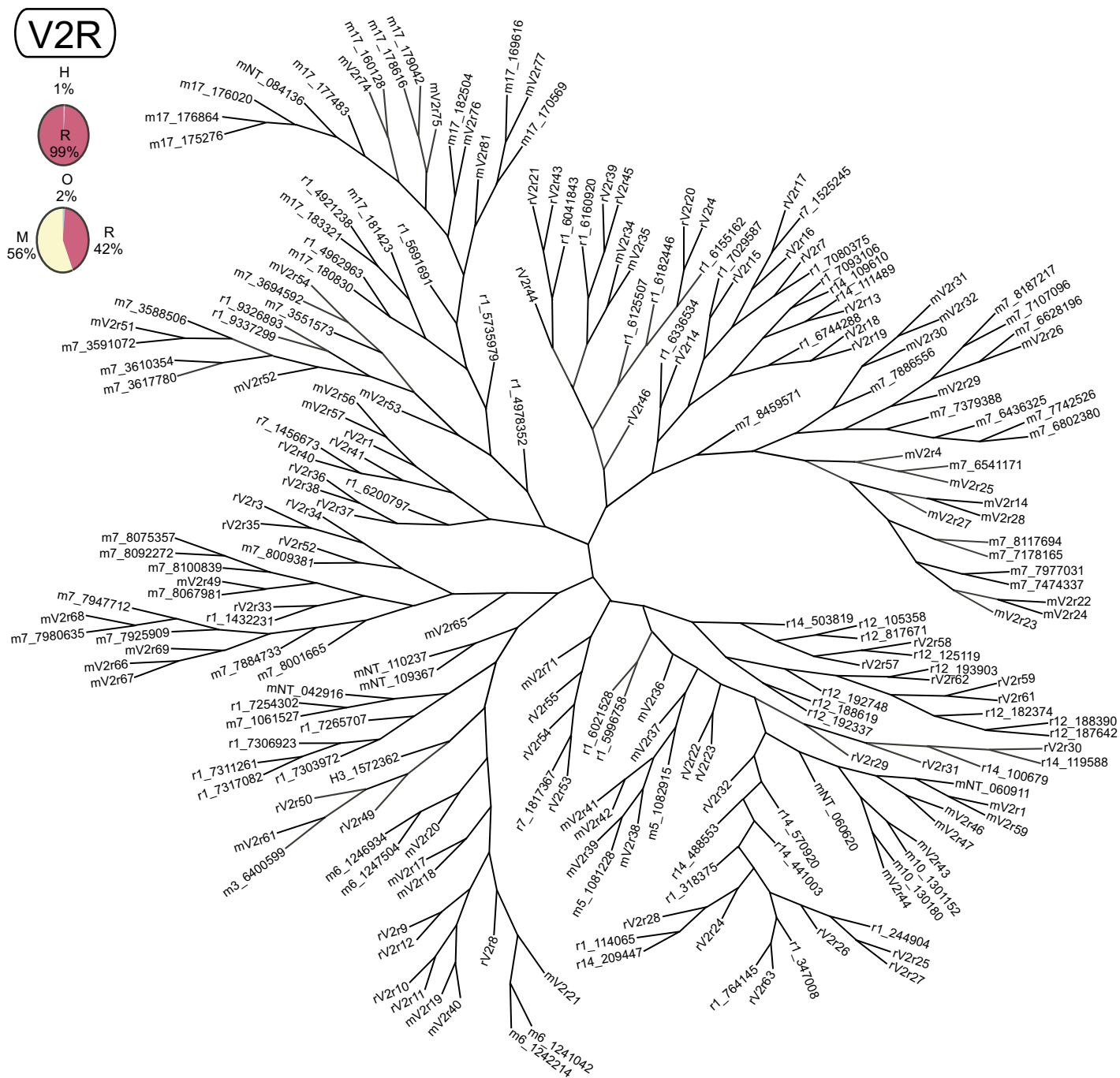

Supplement: Additional file 6 — Phylogenetic tree of the vomeronasal type 2 receptors (V2Rs). The figure shows the consensus trees of 100 maximum parsimony trees of the human and rat vomeronasal type 2 receptors (V2Rs). The first pie chart above the tree shows the proportions of human-rat one-to-one orthologues (O), human specific (H) and rat specific (R) members. The second pie chart from the top displays the proportions of rat-mouse one-to-one orthologues (O), rat specific (R) and mouse specific (M) members. [file 1471-2164-8-338-S6.pdf]

OR

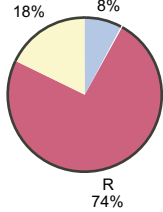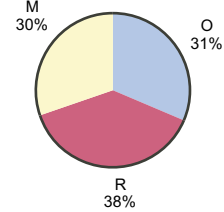

-Class I

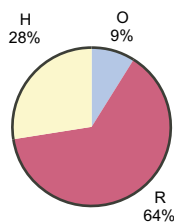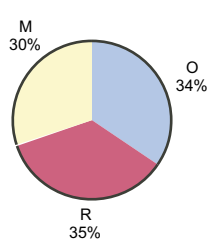

-Class II

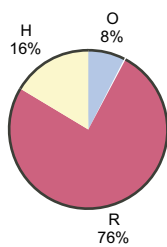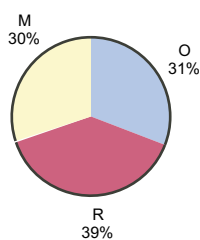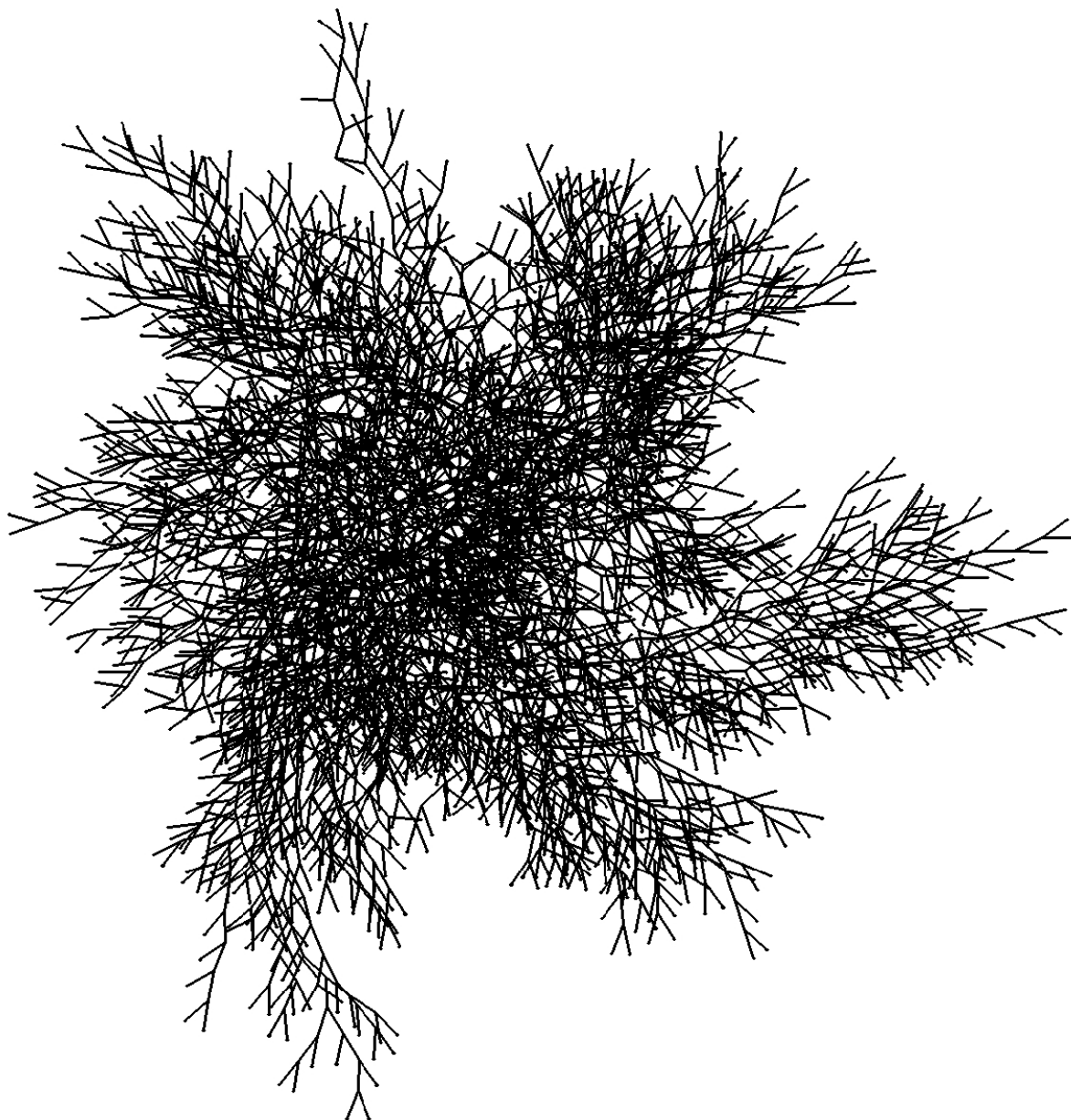

Supplement: Additional file 7 — Phylogenetic tree of the olfactory receptors (ORs). The figure shows the consensus tree of 10 neighbor joining trees of the human, rat and mouse olfactory receptors. The pie charts to the left show the proportions of human-rat one-to-one orthologues (O), human specific (H) and rat specific (R) members. The pie charts to the right display the proportions of rat-mouse one-to-one orthologues (O), rat specific (R) and mouse specific (M) members. The pie charts on the top give the proportions of one-to-one orthologues for all ORs, whereas the charts below contain the same information for the Class I and Class II subsets, respectively. [file 1471-2164-8-338-S7.pdf]
